# Pain management during labor: use of intermittent drug delivery devices for improvement of obstetric and neonatal outcome and reduction of healthcare burden: A large non-inferiority randomized clinical trial

**DOI:** 10.1186/s44158-021-00003-w

**Published:** 2021-09-01

**Authors:** Laura Rinaldi, Anna Maria Ghirardini, Raffaella Troglio, Valentina Bellini, Lara Donno, Susanna Biondini, Emanuela Biagioni, Marco Baciarello, Elena Bignami, Massimo Girardis

**Affiliations:** 1 Anesthesia and Intensive Care Unit, University Hospital of Modena, L.go del Pozzo 71, 41125 Modena, Italy; 2 Anesthesiology and Critical Care Division, Department of Medicine and Surgery, University of Parma, Viale Gramsci 14, 43126 Parma, Italy

**Keywords:** Labor pain, Intermittent epidural analgesia, Patient controlled epidural analgesia, Delivery

## Abstract

**Background:**

Automated continuous epidural administration of local anesthetics provides a more stable analgesic block with decreasing of healthcare staff compared to manual boluses administration (TOP-UP) but is associated to high rate of operative vaginal delivery. We hypothesized that the use of programmed intermittent automated boluses (PIEB) is able to provide a good quality of analgesia and decreasing of anesthesiologic workload without increasing the rate of instrumental vaginal birth in comparison with TOP-UP technique. Laboring nulliparous woman aged between 18 and 46 years were randomized to epidural analgesia with 0.0625% levobupivacaine and sufentanil administered by PIEB or by TOP-UP techniques. Primary outcome was instrumental vaginal delivery rate and secondary outcomes were quality of analgesia, total and time-related drugs doses used, motor block, newborn outcome, and anesthesiologic workload.

**Results:**

Six hundred twenty-nine were randomized, and 628 were included in the intention-to-treat analysis. The rate of instrumental vaginal delivery was similar in the PIEB and TOP-UP groups (13.2% vs 9.7%, OR 1.4 95% CI 0.8 to 2.5; *p* 0.21). There was no difference between groups regarding mode of delivery (cesarean section vs vaginal birth), newborn outcome, and motor block. Patients in the PIEB group received more total and time-related drugs doses and a better quality of analgesia. Anesthesiological workload was significantly reduced in the PIEB group.

**Conclusions:**

Our study demonstrated that epidural anesthesia with programmed intermittent epidural boluses by an automated device provides an effective and safe management of labor analgesia with improvement of pain control and sparing of man workload compared to manual top-up protocols.

## Background

Health and welfare of women in labor and newborns represents a priority commitment all over the world. In this context, pain relief during labor is an important issue considering that more than 60% of nulliparous delivering women quantifies labor pain from severe to intolerable [1]. Epidural analgesia (EA) has been recognized as the most effective technique to control labor pain, and it is used consistently in numerous countries [2, 3]. Although robust evidences indicate that EA does not increase the incidence of cesarean sections, a possible increase of operative vaginal delivery and, thus, of maternal and newborn risk has been observed in patients with EA and mainly attributed to large doses of local anesthetic with the consequent onset of sacral motor block [1, 3–5].

Intermittent drugs bolus (top-up) was the first technique for EA in labor. Although it provides an adequate pain control even in patients with prolonged labors or in the second stage of labor [6], top-up technique may transiently expose the patient to unsatisfactory analgesia and requires the constant presence of the anesthetist in the labor room. Continuous infusion of local anesthetic, when compared to top-up technique, may provide a more stable analgesic block and reduction of the anesthesiologist workload [7]. Therefore, it progressively became the most used technique worldwide, particularly in settings with high volumes of EA. Despite potential advantages, continuous infusion seems to be associated with an increased number of operative deliveries, ranging from 20% up to 50% [8, 9]. A systematic review by the Cochrane group [3] showed that the relative risk of assisted vaginal birth is increased (1.42, 95% CI 1.28 to 1.57) by using continuous infusion compared to the top-up protocols. As neonatal outcome seems to be related to the instrumental delivery rather than to the use of EA [10], the risk benefit ratio of EA with continuous infusion of local anesthetic should be carefully evaluated during labor analgesia, especially in patients at high risk for operative delivery.

To overcome the risks related to continuous infusion, the administration of intermittent boluses by automatic infusion pumps has recently been proposed with promising results [11–13]. Unfortunately, no data are available for the safety comparison between intermittent doses performed by anesthetists (top-up) or by automated devices, associated or not to patient-controlled administration. Our study is aimed to determine whether the use of an automated intermittent bolus device compared to standard manual top-up methods may prevent the increase of instrumental deliveries while maintaining a satisfactory level of analgesia and allows a reduction of anesthesiologic workload in the delivery room.

## Methods

This multi-site, open label, randomized controlled trial (NCT02710877) is based on intention-to-treat analysis and non-inferiority two-arm design with one-to-one allocation. Anesthesiologic units of the University Hospitals of Modena and Parma, with around 3000 deliveries per year, participated in the study. The Carpi General Hospital was at first included in the study as the third center, but they were unable to enroll patients due to low human resources during the study period (December 23, 2014, to December 27, 2017).

Ethical committees of all centers approved the study. Written informed consent was obtained from all subjects.

Healthy, nulliparous, term women with singleton, vertex pregnancies in spontaneous labor were eligible to participate in the study. Women with any disorder of pregnancy, breech or multiple gestation, or who were unable to perform motor block evaluation tests were excluded from the study. Women who met the above criteria were requested to give written informed consent to participate. The parturient was admitted to the study if her baseline pain score, assessed at the peak of the contraction, was 5 or more on an 11-point numerical rating scale (NRS) (where 0 was “no pain” and 10 “worst pain imaginable”), if cervical dilation was < 5 cm, and if she had not received oxytocin before epidural analgesia.

Epidural analgesia was initiated in the sitting position at the lumbar 3–4 or 2–3 interspaces. The epidural space was identified using the loss of resistance to saline technique (1–2 mL) with a 17-gauge Tuohy epidural needle. A closed-end, multiorifice epidural catheter was inserted 3 to 4 cm into the epidural space through the Tuohy needle and secured. A 5-ml 0.0625% levobupivacaine test dose was administered. The parturients received an initial epidural loading dose consisting of 0.0625% levobupivacaine 15 mL plus sufentanil 10 mcg. Before epidural catheter placement, a sequentially numbered opaque envelope containing the group assignment (computer-generated random-number sequence) was opened by the researcher who set up the epidural catheter.

Parturients were randomized to receive one of the following regimens for the management of analgesia: manual anesthesiologist bolus (group TOP-UP) or programmed intermittent epidural boluses (PIEB) by an automated device (group PIEB). In the control group, the anesthesiologist administered 0.0625% levobupivacaine 15 ml with sufentanil 5 mcg every 90 min or on patient request, as on standard top-up protocols. The interval between manual boluses could change according to rescue drug administration and clinical setting. The PIEB pump was programmed to deliver 0.0625% levobupivacaine with sufentanil 0.4 mcg/mL, 10 mL every 75 min, beginning 75 min after the administration of the initial epidural loading dose. Besides, the pump for PIEB was programmed to deliver 5-mL patient-activated boluses (of the same drug mixture with a lockout interval of 10 min, and a per hour maximum volume of 20 mL). Patients were instructed, before or immediately after the epidural catheter placement, on how to use the PIEB pump remote and to push the button whenever they felt uncomfortable. If the parturient still felt pain after manual boluses or after activating the PIEB bolus twice in a 20-min period, an anesthesiologist administered additional manual incremental boluses of 5-mL levobupivacaine 0.125% until the NRS score was < 5. The epidural infusions (TOP-UP or PIEB) were continued through the second stage of labor until the delivery of the fetus.

Data recorded for each subject included demographic characteristics, labor data, analgesia and motor block evaluation, mode of delivery, and neonatal scores. The total infused volumes was obtained from the infusion pump data recording system. Similarly, in the TOP-UP group, the number and total volume of manual boluses were recorded on a specific clinical chart. The use of local anesthetic for episiotomies or instrumental assisted vaginal delivery analgesia was not included in the total drug calculation. NRS score for pain and motor function were evaluated every 60 min beginning 30 min after the epidural injection during the first stage of labor and at full cervical dilation (before pushing). The degree of motor block was assessed in both lower extremities using the Bromage modified score, whereby 1 = no block and 4 = complete block. Neonatal outcome was evaluated at birth through a three-point score, resulting from the following positive items: 5 min Apgar score < 8, presence of fetal acidosis (umbilical artery pH< 7.15 or base deficit < − 10) and need for resuscitation maneuvers, i.e., positive pressure ventilation or active airways management. The anesthetist work time was the sum of the minutes spent in labor room for catheter placement and patient management until birth. Decision to proceed toward operative delivery was set by obstetric team in charge who was not involved in the study management.

## Endpoints

The rate of instrumental assisted vaginal deliveries on the total vaginal deliveries was the primary endpoint. Secondary outcomes were the rate of cesarean sections, the rate of patients with average NRS < 5 during analgesia, the total and the time-related dose of local anesthetic, the occurrence of motor block with Bromage scale> 1, the neonatal score, and the anesthetist working time.

## Sample size and statistical analysis

To detect a clinically significant 7% increase in the rate of instrumental assisted deliveries between the two groups given a pre-study instrumental delivery rate of 13% in the trial centers, a statistical power of 80%, a two-tailed alpha error of 5% and an expected 10% of withdrawal rate, and a sample size of 450 parturient per group was needed. Unfortunately, the lack of enrollments by the Carpi Hospital Center caused a recruitment rate lower than expected. Despite the 1-year prolongment of the study period in the Modena Hospital Center, the study ended without reaching the calculated sample size because of over budget.

Data were primarily analyzed according to the intention-to-treat principle. The primary outcome, instrumental delivery rate, was presented as frequencies and compared across study groups using a chi-square test for binary data. All categorical data were analyzed using *χ*^2^ analysis. The normality of quantitative data was assessed using the Kolmogorov-Smirnov test. Paired Student’s *t* test, Fisher exact test, ANOVA, and Mann–Whitney *U* test tests were used to compare means. Data are presented as mean and standard deviation (SD), percentage of group total, or median with interquartile range, as appropriate. We also estimated the relative risks using the TOP UP group as reference. The significance level was set at 0.05 for all analyses. IBM SPSS Statistics version 20.0 (SPSS Inc., Chicago, IL, USA) was used for sample size calculation, randomization, and analysis.

## Results

**Fig. 1:**
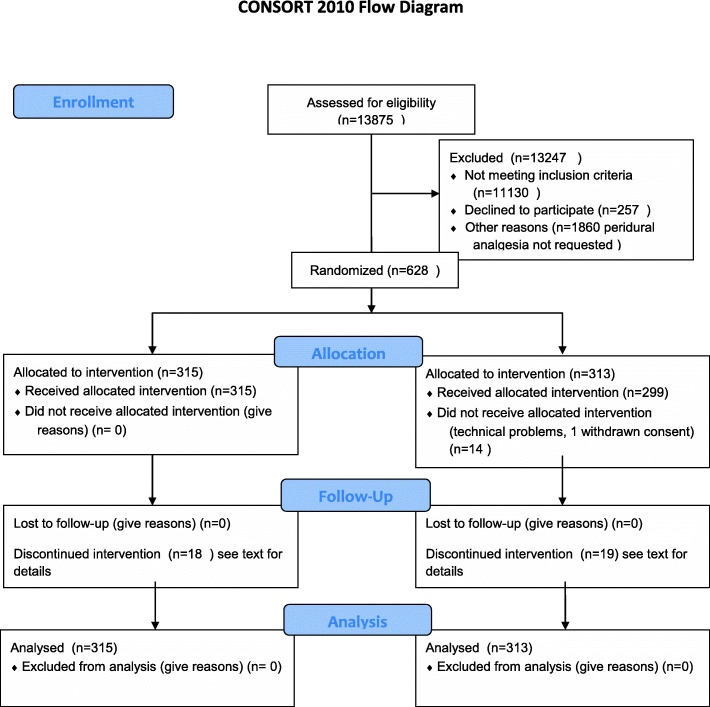
CONSORT 2010 flow diagram

A total of 13,875 patients were assessed for eligibility from December 2014 to December 2017, and a total of 629 patients were randomized, 315 in the TOP UP group and 314 in the PIEB group, but 628 were analyzed because of one withdrawn consent. Thirty-seven of the cases were withdrawn because of protocol infraction (25) or catheter dislocation (12), and thirteen cases in PIEB group encountered technical problems with the pump. See the CONSORT diagram for details (Fig. 1).

The demographics and obstetric characteristics were similar between the two study groups (Table 1). In the PIEB group, the rate of instrumental assisted delivery (primary outcome) as well as the rate of cesarean section did not differ from the rates observed in the TOP-UP group (Table 2). The PIEB group showed a better control of pain throughout the study period with lower average NRS and lower rate of patients requiring rescue doses of local anesthetics compared to the TOP-UP group. The dose per minute of analgesia and the total amount of local anesthetic were higher in the PIEB group (Table 2). Motor block with Bromage score ≥ 2 was observed only in 3 patients (1%) of the PIEB group (*p* 0.08). The anesthetist workload was significantly reduced by the PIEB protocol compared to the TOP-UP (77.1 ± 35.2 vs 87.6 ± 46.6 min; *p* 0.003). No differences were observed between groups in neonatal outcome (*p* 0.935) (Table 2).

**Table 1:** Patients demographics and obstetric characteristics

	TOP-UP (*n* = 315)	PIEB (*n* = 313)	*p*
Maternal age; years, mean (SD)	32.2 (5.2)	31.7 (5.3)	0.26
Pre-pregnancy body mass index; kg/m^2^ mean (SD)	25.6 (4.7)	26.1 (4.2)	0.23
Labor induction; *n* (%)	40 (12.7)	30 (9.7)	0.21
Basal NRS; median-IQR	10 (8–10)	10 (8–10)	0.97

*NRS* numeric rate scale, *IQR* interquartile range

**Table 2:** Primary and secondary outcomes

	TOP-UP (*n* = 315)	PIEB (*n* = 313)	*p*	RR (95% CI)
Instrumental assisted delivery; *n* (%)	27 (10.5)	36 (13.6)	0.28	1.3 (0.8–2.3)
Cesarean section; *n* (%)	58 (18.4)	48 (15.3)	0.30	0.8 (0.5–1.2)
Total operative delivery; *n* (%)	85 (27)	84 (26.8)	0.97	0.99 (0.7–1.4)
NRS average; mean (SD)	4.8 (1.8)	4.0 (1.9)	< 0.0001	
Parturients with mean NRS < 5; *n* (%)	146 (47.2)	209 (67.7)	< 0.0001	0.43 (0.3–0.59)
Parturients requiring rescue drug; *n*; (%)	156 (49.5)	90 (28.8)	< 0.0001	0.4 (0.3–0.6)
Total dose of rescue levobupivacaine; mg, mean (SD)	7.8 (12.3)	2.6 (5.3)	< 0.0001	
Dose of levobupivacaine per minute of analgesia; mg/min, mean (SD)	0.085 (0.05)	0.099 (0.05)	0.001	
Total dose of levobupivacaine; mg, mean (SD)	33.4 (22.2)	45.3 (23.9)	< 0.0001	
Neonatal score				
0 point, *n* (%)	258 (81.9)	258 (82.4)	0.988	
1 point, *n* (%)	48 (15.2)	45 (14.4)		
2 points, *n* (%)	8 (2.5)	8 (2.6)		
3 points, *n* (%)	1 (0.3)	2 (0.6)		

*NRS* numeric rate scale, *IQR* interquartile range

## Discussion

This large randomized non-inferiority trial demonstrated that labor analgesia delivered with programmed intermittent epidural boluses by an automated device does not increase the rate of operative vaginal delivery and cesarean sections compared with the manual top-up anesthesia. In addition, automated programmed intermittent analgesia provided a better average pain control by using larger total doses of low concentration local anesthetics and fewer patients requiring rescue doses compared to conventional manual top-up protocol with reduction of anesthetist’s workload and similar neonatal outcome.

The obstetric benefits provided by the PIEB technique in comparison to epidural continuous infusion have been previously reported [11–14]. However, in those trials, the rate of spontaneous vaginal delivery ranged between 30 and 70% of total births [12, 14], whereas in our trial, the PIEB protocol was comparable to top-up protocol with > 70% of spontaneous vaginal deliveries. Interestingly, the above trials reported lower average pain scores than ours, while in trials focused on motor blockade and obstetric outcome, the average pain score was similar to our data [6, 7, 13, 15–17]. The NRS threshold of 5 used in our trial was the common threshold in the participating centers before the trial and discussed with parturients during consent acquisition for the procedure.

One of the goals of modern labor analgesia is reducing motor blockade in order to obtain a “walking analgesia” by accepting even higher pain intensity. In our study, motor block was never observed in the top-up group and rare also in the PIEB group (1%), demonstrating that this automated administration device can preserve women ability of moving and assuming whatever position during labor and delivery. In literature, the incidence of motor blockade is usually significantly higher, ranging between 30 and 50% of labor epidural analgesia [13, 18–22]. Although Tixier and colleagues observed no differences in motor block by comparing levobupivacaine 0.0625% and 0.125% [23], there is a strong view that the risk of motor block increases with increasing concentration of local anesthetics and the use of continuous infusion [13]. The use of low concentration bupivacaine and PIEB or manual top-up could explain the very low incidence of motor block in our trial.

In general, the use of analgesics does not seem to influence fetal oxygenation, neonatal pH, or 5-min Apgar scores that are frequently impaired by operative delivery [10, 24]. This association is confirmed by our study that provided similar results in terms of safety for neonatal outcome between the two protocols despite differences in the total amount of bupivacaine administration.

The recent introduction of labor epidural analgesia (EA) in the Italian Essential Levels of Health Care administered by the Public Health System will produce an increase in the request of labor analgesia with increased workload for both anesthetist and obstetric teams. In many Italian hospitals, the manual administration of epidural boluses still constitutes the reference technique for the maintenance of the labor EA, according to an operative choice made when the number of requests was limited. Nevertheless, the incidence of operative delivery with the use of top-up is currently variable, from 35% [12] to 14%, with low concentrations of local anesthetic [14]. The impact of an automated regular bolusing device on human resources has never been studied before our trial. Institutions where labor epidural analgesia is delivered by manual analgesic boluses, frequent in our country, need high man workload that could lead to poor pain relief due to irregular administration during multiple contemporary labor analgesia or different emergencies. Our findings confirmed that PIEB represents a welcome improvement to enhance the quality and safety of analgesia with a clear decrease in manpower utilization.

Our study has several limitations. First of all, the study was stopped before the achievement of the planned sample size. Therefore, despite the multicentric design and large sample size, the results of the trial should be considered with caution because early termination of the trial may have exaggerated the effect size. In fact, by assuming the same rate of assisted delivery observed, if the trial had achieved the planned sample size (405 patients per group considering 10% of drop-out), the estimation of the study 95% CIs ranged between − 1% and 7.6% that do not entirely rule out the planned difference for non-inferiority of 7%. Moreover, we limited our trial to nulliparous patients without oxytocin labor induction to homogenize the duration of analgesia avoiding dystocic and highly risky labors. Therefore, our results cannot be extended to other laboring conditions, like multiparous women or oxytocin induced labor. Although the collection of pain intensity was planned at specific time points during the study, these were often not respected because of maternal requests of top-up boluses in the control group. The data are limited also in the location and number of episodes of breakthrough pain, which were not collected per se, but only in average NRS scores.

In conclusion, the PA-RER trial demonstrated that patient controlled epidural anesthesia with programmed intermittent epidural boluses by an automated device provides an effective and safe management of labor analgesia with improvement of pain control and sparing of man workload compared to manual top-up protocols. Although further trials are needed for definitive confirmation of our results, we believe that this method should be considered as a valuable alternative to manual top-up protocols in centers with multiple patients at the same time and to continuous infusion protocols in centers with high rate of operative deliveries.

## Data Availability

The datasets used and analyzed during the current study are available from the corresponding author on reasonable request.
