# Correction to: Pain management during labor: use of intermittent drug delivery devices for improvement of obstetric and neonatal outcome and reduction of healthcare burden: a large non-inferiority randomized clinical trial

**DOI:** 10.1186/s44158-021-00011-w

**Published:** 2021-10-25

**Authors:** Laura Rinaldi, Anna Maria Ghirardini, Raffaella Troglio, Valentina Bellini, Lara Donno, Susanna Biondini, Emanuela Biagioni, Marco Baciarello, Elena Bignami, Massimo Girardis

**Affiliations:** 1 Anesthesia and Intensive Care Unit, University Hospital of Modena, L.go del Pozzo 71, 41,125 Modena, Italy; 2 Anesthesiology and Critical Care Division, Department of Medicine and Surgery, University of Parma, Viale Gramsci 14, 43,126 Parma, Italy

**Correction to: J Anesth Analg Crit Care 1, 2 (2021)**

**https://doi.org/10.1186/s44158-021-00003-w**

Following publication of the original article [1], the authors identified that the given names and family names of all authors were swapped.

The author group has been updated above and the original article has been corrected.
